# Morpho-Functional Evaluation of Full-Thickness Macular Holes by the Integration of Optical Coherence Tomography Angiography and Microperimetry

**DOI:** 10.3390/jcm9010229

**Published:** 2020-01-15

**Authors:** Daniela Bacherini, Maria Cristina Savastano, Francesco Dragotto, Lucia Finocchio, Chiara Lenzetti, Alice Bitossi, Ruggero Tartaro, Fabrizio Giansanti, Francesco Barca, Alfonso Savastano, Tomaso Caporossi, Lorenzo Vannozzi, Andrea Sodi, Marino De Luca, Francesco Faraldi, Gianni Virgili, Stanislao Rizzo

**Affiliations:** 1 Department of Neurosciences, Psychology, Drug Research and Child Health Eye Clinic, University of Florence, AOU Careggi, 50139 Florence, Italy; francesco.dragotto@gmail.com (F.D.); luciafinocchio@gmail.com (L.F.); chiaralenze@gmail.com (C.L.); alicebitossi@yahoo.it (A.B.); ruggero.tartaro@gmail.com (R.T.); fabrizio.giansanti@unifi.it (F.G.); barcaf@hotmail.com (F.B.); asavastano21@gmail.com (A.S.); tomaso.caporossi@me.com (T.C.); lorenzo.vannozzi@tin.it (L.V.); andreasodi2@gmail.com (A.S.);; 2 Livorno Hospital, Eye clinic, 57124 Livorno, Italy; oculistadeluca@gmail.com; 3 Centro Italiano Macula, 00195 Rome, Italy; mariacristina.savastano@gmail.com; 4 Torino, Eye clinic, ASL Torino 5, 10024 Turin, Italy; faraldi@retinatorino.it

**Keywords:** **OCT** Angiography, microperimetry, full-thickness macular hole

## Abstract

(1) Objective: To use optical coherence tomography angiography (OCTA) and microperimetry (MP) to evaluate the correlation between retinal structure and function in patients with idiopathic, full-thickness macular holes (FTMHs) (2) Methods: This prospective, observational study included 11 eyes of 10 patients with FTMHs evaluated before surgery using OCTA and MP. MP sensitivity maps were superimposed and registered on slabs corresponding to superficial capillary plexus (SCP) and deep capillary plexus (DCP) on OCTA, and on the outer plexiform layer (OPL) and the Henle fiber layer (HFL) complex in en face OCT. On these maps, mean retinal sensitivity was calculated at 2° and 4°, all centered on the FTMH. Cystic cavity extension was assessed on the slab corresponding to the OPL + HFL complex in en face OCT and DCP in OCTA using the Image J software (Version 1.49v; National Institutes of Health, Bethesda, MD, USA); (3) Results: Absolute scotomas were observed corresponding to the FTMH. Additionally, rings of relative scotoma in the perilesional area were detected and correlated to the cystic spaces on en face OCT and OCTA. There was a significant correlation between reduced retinal sensitivity at 2° and 4° diameters around the FTMH and the extension of cystic areas (*p* < 0.01). There was a significant correlation between the extension of cystic cavities and BCVA (*p* < 0.01). (4) Conclusions: Morpho-functional analysis of FTMH using OCTA and MP, and the correlation between vascular abnormalities and impaired retinal sensitivity, may provide new, useful information. This integrated evaluation of FTMH may be useful to determine the function–structure correlation before and after vitreoretinal surgery, in order to gain a better understanding of the functional consequences induced by the morphological alterations, assessing outcomes in a more objective way, and potentially adding new surgical prognostic factors.

## 1. Introduction

A full-thickness macular hole (FTMH) is a foveal defect involving all the neural retinal layers, from the internal limiting membrane to the outer segment of the photoreceptor layer. Vitrectomy with internal limiting membrane (ILM) peeling provides anatomical closure in the majority of cases (90–95%) [1]. Several studies have reported preoperative factors that are predictive of visual outcomes following vitrectomy for FTMH [2,3,4,5]. These factors include the stage and size of a MH [2], duration of symptoms [3], preoperative visual acuity [4], retinal sensitivity, and status of fixation [5]. Optical coherence tomography (OCT) characteristics may also be predictive factors, including the minimum diameter of the MH, the basal hole diameter, the hole form factor, the MH index, and inner segment/outer segment junction defect length [6]. However, these factors do not accurately predict postoperative visual outcomes. Although both the duration of the symptoms and the hole size are widely accepted as being helpful for predicting visual outcomes following MH surgery, the results for these predictors vary across studies. In fact, visual outcomes are often worse than expected despite successful FTMH surgery.

The recent introduction of OCT Angiography (OCTA) [7] permits a noninvasive evaluation of retinal vasculature in different chorioretinal diseases. Currently, OCTA is most commonly used to evaluate neovascular diseases [8,9], occlusive retinal diseases [10,11], or diabetic retinopathy [12,13]. However, it can also be used in surgical vitreomacular pathologies to evaluate vascular changes and the effects of macular surgery on retinal microvasculature in a noninvasive way [14,15].

Fundus microperimetry is a precise, repeatable, morphology-related modality that provides a functional evaluation of retinal sensitivity [16,17]. Few studies that have reported a correlation between macular sensitivity (MS) and foveal thickness and best corrected visual acuity (BCVA) following vitreoretinal surgery for epiretinal membranes [18]. However, studies have confirmed that MS is a significant prognostic factor for postoperative functional outcomes [19].

The correlation of retinal sensitivity measured using microperimetry with structural findings from en face OCT images and vascular findings from OCTA could be useful when evaluating the surgical treatment for FTMH.

The purpose of this study is to correlate the retinal sensitivity of patients affected by FTMH with the retinal architecture evaluated by OCT and OCT-A imaging.

## 2. Experimental Section

### 2.1. Study Design

This observational cross-sectional study evaluated 11 eyes affected by idiopathic FTMH in 10 patients at baseline before vitrectomy and peeling of epiretinal membranes and inner limiting membrane (ERM-ILM), using OCT, OCTA and microperimetry.

This study adhered to the tenets of the current version of the Declaration of Helsinki (52nd WMA General Assembly, Edinburgh, Scotland, October 2000), and written informed consent was obtained from all patients prior to participation in the study. Approval from The Institutional Review Board/Ethics Committee was obtained.

The inclusion criterion was the presence of a FTMH with cystoid spaces that were well detected in the inner nuclear layer (INL) and the OPL + HFL complex using structural B-scan OCT. Only patients with OCT and OCTA images of adequate quality were included.

Patients were excluded if they had significant media opacities and concomitant diseases such as diabetic retinopathy, vein or artery occlusion, glaucoma, or posterior uveitis.

### 2.2. Baseline Evaluation

All patients underwent a complete ophthalmic examination, including medical and ocular history, measurement of best corrected visual acuity (BCVA) using ETDRS charts in decimal, intraocular pressure measurement, slit-lamp biomicroscopy of the anterior segments, dilated fundus examination, axial length measurement with no-contact partial coherence laser interferometry (IOL Master, version 3.01; Carl Zeiss Meditec, Germany), OCTA (RS-3000 Advance 2 spectral domain OCT; NIDEK Co. Ltd., Gamagori, Japan) and microperimetry (MP-3; NIDEK Co. Ltd., Gamagori, Japan). All patients underwent measurement of BCVA and OCT at three months after surgery.

### 2.3. SD-OCT Angiography with RS3000 Advance 2

OCTA RS3000 Advance 2 uses an 880 nm wavelength with a scanning speed of 85,000 A Scans/s.

This system is based on the CODAA (complex OCT-signal differential analysis angiography) algorithm, which uses blood flow as the intrinsic contrast, analyzing both phase and amplitude variations of the OCT signal to detect blood flow motion and render OCT-A scans.

OCTA scans were acquired following a standardized protocol. We performed 3 × 3 mm (256 × 256 scan points) scans centered on the fovea based on a real-time SLO image. An active eye-tracking system permitted vertical, horizontal, and cyclotorsional compensation during image acquisition. All B-scans were averaged eight times.

**Figure 1:**
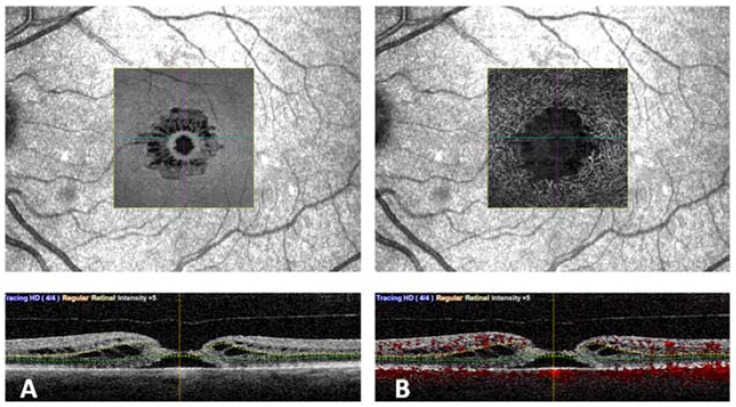
An eye with full thickness macular hole analyzed using en face optical coherence tomography (OCT) (**A**) and OCT angiography (**B**). In the lower part the corresponding segmentation is visible.

Vascular retinal layers were visualized and segmented in the superficial capillary plexus (SCP), the deep capillary plexus (DCP), and the choroid. Images were reviewed by two investigators (DB and FD) for segmentation accuracy. Automated segmentation of SCP, DCP, and CC was performed. (Figure 1). Manual adjustment of the segmentation was performed in cases of severe alteration of the macular cytoarchitecture.

**Figure 2:**
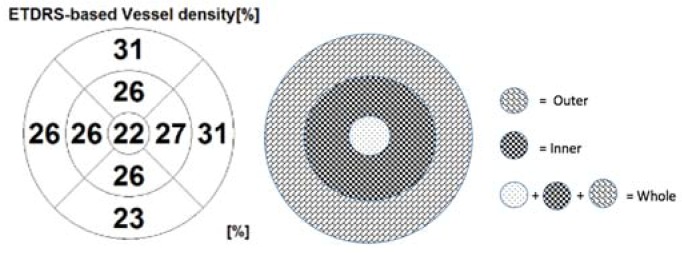
The ETDRS-based vessel density [%], with division of the macular area into the nine ETDRS subfields (on the left). On the right, a scheme showing the rings centered around the fovea. The fovea is defined as the area within the central 1-mm ring of the Early Treatment Diabetic Retinopathy Study (ETDRS) grid. The surrounding ring with an inner diameter of 1 mm and an outer diameter of 3 mm is considered as the inner ring. The ring with an inner diameter of 3 mm and an outer diameter of 6 mm is considered as the outer ring. The whole ring includes the fovea and the inner and outer rings.

The default RS-3000 Advance 2 OCTA AngioScan software was used to evaluate the vessel density (VD) (ETDRS-based vessel density [%]) of the SCP and DCP. Vascular density was defined as the percentage of the total area occupied by vessels. Vessel densities of the SCP and DCP were automatically calculated by the software on OCTA 3 × 3-mm volume scans in the whole foveal and the inner and outer retina (Figure 2).

On OCTA, the mean area of window defect (the area of peak of flow on the choroidal slab due to a lack of the neuroepithelium) was measured (mm^2^).

OCTA scans of the DCP were analyzed and used to measure the radial hyporeflective cavities (areas of no flow), since in the DCP, the wider extension of perifoveal cystic spaces can be identified.

En face OCT scans from the OPL + HFL complex were analyzed. For each layer, the scan with the highest quality was selected. This scan was used to measure the area of the hyporeflective cystic spaces in the coronal plane.

The extension of cystic cavities was measured using Image J software (Version 1.49v, Wayne Rasband; National Institutes of Health, Bethesda, MD, USA. http://imagej.nih.gov/ij), as previously described [20]. The resulting binary images identified hyporeflective spaces as areas that were automatically calculated by particle analysis function; these were included for the statistical analysis. The area (mm^2^) of the cystoid cavities in the OPL+ HFL using en face OCT and in the DCP from OCTA underwent statistical analyses.

### 2.4. Microperimetry with MP-3

Microperimetry MP-3 was used to quantify macular sensitivity.

For the purpose of this study, the following microperimetry variables were used: a red circle fixation target 1.0° in diameter; a white, monochromatic background set at 31.4 asb; Goldman III stimulus size, with a 200-millisecond projection time and a 4–2 fast strategy. The manufacturing default for the macula-8° with 45 spots centered on the fovea region was used for all cases.

As both pieces of equipment can be managed by the same filing software, the Nidek Navis Ex platform (NIDEK Co., LTD, Gamagori, Aichi, Japan), precise registration between OCT-A and microperimetry exams can be performed.

Specifically, the SLO image, that acts as a fundus morphological reference on the OCT-A scans, and the color fundus retinography, that acts as a morphological reference on microperimetry exams, can be registered performing image scaling and rotation, thus allowing precise overlay between any of the OCT-A slabs and the corresponding functional retinal sensitivity values given by the microperimetry.

The software of the microperimeter permitted exact coregistration and overlay of the microperimetry results onto the slabs corresponding to the superficial capillary plexus and deep capillary plexus using OCTA, and the outer plexiform layer (OPL) and the Henle fiber layer (HFL) complex using en face OCT.

On these maps, mean retinal sensitivity was calculated at 2° and 4°, both of which were centered on the macular hole. Absolute scotomas were defined as loci where no response was registered, even at the highest stimulus (0 dB).

### 2.5. Statistical Analysis

The data were analyzed with PRISM Software (Version 6.0; GraphPad Software, La Jolla, CA, USA). Visual acuity data were converted to LogMAR values for statistical analysis. The association between the total cavity area and retinal sensitivity was evaluated with the Pearson coefficient correlation for the area within the inner retina. The area within this layer was also correlated to BCVA. The vascular densities and the peak signal measured using OCTA in the choroidal slab were plotted against 2° and 4° retinal sensitivity maps in eyes with FTMH. *p* values less than 0.05 were considered statistically significant.

## 3. Results

The study sample included 11 eyes of 10 patients (7 females and 3 males) affected by idiopathic FTMH. The mean age of the study sample was 59.2 ± 3.7 years. The mean baseline BCVA was 0.8 ± 0.25 LogMAR. The mean axial length was 23.31 ± 0.69 mm. There was one eye with stage 2 FTMH, five with stage 3, and five with stage 4. There were six eyes with macular adhesion or traction. The mean macular hole diameter was 423.72 ± 197.93 μm. The mean postoperative was 0.5 ± 0.4 LogMAR BCVA at three-month follow-up.

The mean preoperative VD of the whole SCP was 16.91 ±1.14% and the mean VD of the whole DCP was 15.18 ± 2.92%. The mean VD of the inner SCP was 18.27 ± 1.0% and the VD of the outer SCP 17.63 ± 2.29%. The mean VD of the inner DCP was 14.82 ± 3.31% and the VD of the outer DCP 18.73 ± 4.61%. The mean central retinal sensitivity was 2.4 ± 3.07 dB at 2°, 5.01 ± 4.23 dB at 4°.

Software permitted registration and exact overlay of the microperimetry results onto the slabs corresponding to the deep capillary plexus on OCTA, and the outer plexiform layer (OPL) and the Henle fiber layer (HFL) complex on en face OCT.

**Figure 3:**
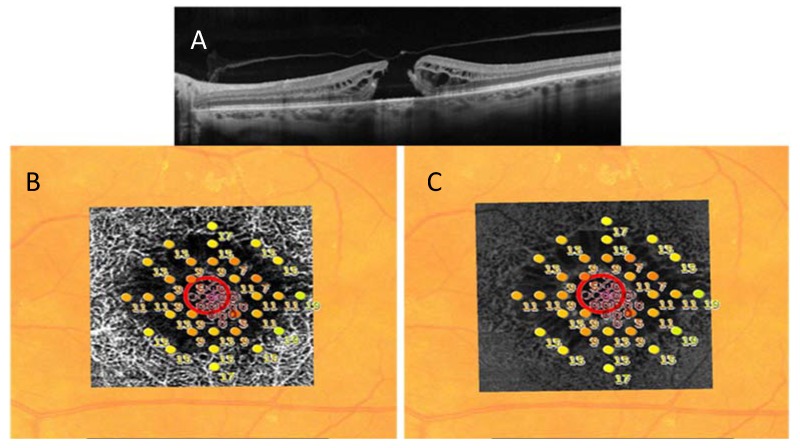
(**A**) The B-scan OCT shows a full-thickness macular hole. (**B**) The central absolute scotoma corresponding to the FTMH and rings of relative scotoma extending in the perilesional area are shown in microperimetry map. These relative scotomas correlate with the cystic alterations detected by OCT angiography at the level of deep capillary plexus (**B**) and by en face OCT in the outer plexiform layer (OPL) and the Henle fiber layer (HFL) complex (**C**).

Absolute central scotomas corresponded to the FTMH, and a ring of relative scotoma in the perilesional areas was identified. This relative scotoma in the overlapped images correlated with the cystic alterations at the level of DCP on OCTA and the OPL + HFL complex on en face OCT (Figure 3).

**Figure 4:**
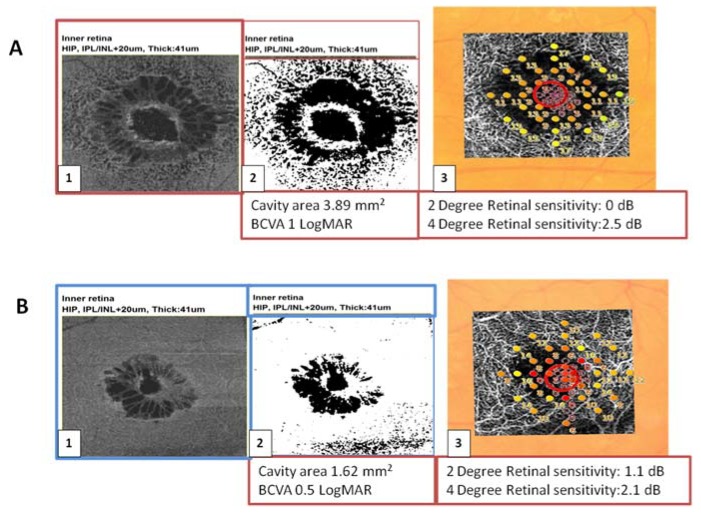
(**A**) 1. En face scan of the OPL + HFL complex of a large MH. 2. The extension of cystic cavities measured on the slab corresponding to OPL + HFL complex assessed by OCT en face, using the Image J software. In the lower part the corresponding cavity area in mm^2^ and the Best Corrected visual Acuity in LogMAR are indicated. 3. Overlay of the microperimetry (2° retinal sensitivity) on the slabs corresponding to the deep capillary plexus on OCTA. (**B**) 1. En face OCT (OPL + HFL complex) of a smaller macular hole. 2. The extension of cystic cavities measured on the slab corresponding to OPL + HFL complex assessed by OCT en face, using Image J software. 3. Overlay of the microperimetry (2° retinal sensitivity) on the slabs corresponding to the deep capillary plexus on OCTA.

The larger MHs with a wider extension of cystic cavities were associated with lower BCVA and a lower 2° and 4° retinal sensitivity compared to the smaller MHs (Figure 4).

**Figure 5:**
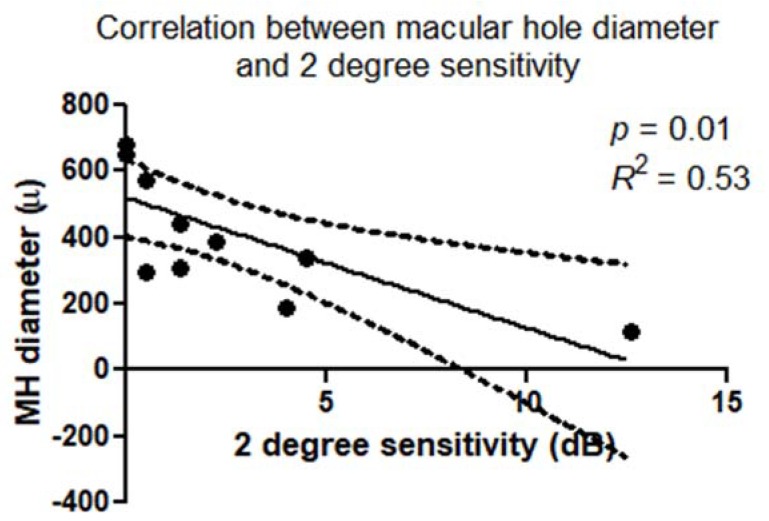
Scatterplot shows the statistically significant correlation (*p* = 0.01; *R*^2^ = 0.53) between macular hole diameter (μm) and microperimetry 2 degree sensitivity (dB).

There was a statistically significant correlation between retinal sensitivity (dB) and macular hole diameter (μm). This correlation was confirmed within a 2° diameter (*p* = 0.01; *R*^2^ = 0.53) (Figure 5) and 4° diameter (*p* = 0.03; *R*^2^ = 0.40) around the MH.

There was a statistically significant correlation between the extension of the cystic cavities and BCVA (*p* = 0.0030; *R*^2^ = 0.642) and between macular hole diameter (μm) and BCVA (*p* < 0.001).

The mean area of the cavities on en face OCT was 2.51 ± 0.89 mm^2^, and the mean corresponding area of no flow on OCTA was 2.52 ± 1 mm^2^, i.e., without statistical difference (*p* > 0.05).

**Figure 6:**
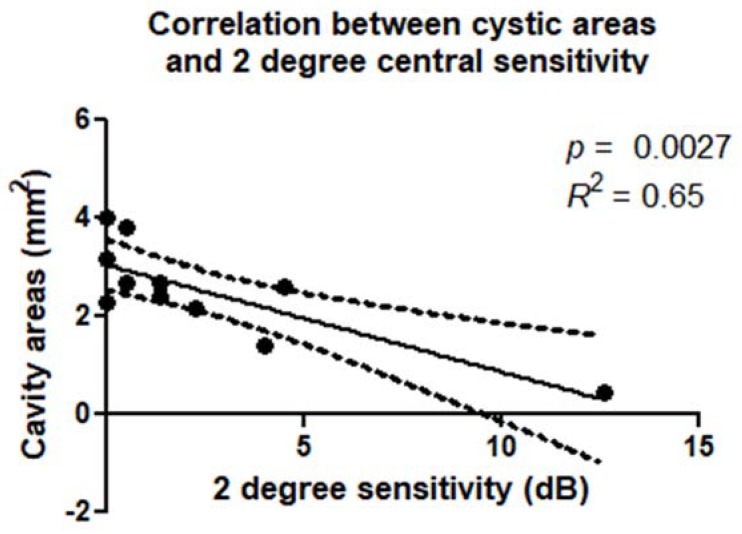
Scatterplot shows the statistically significant correlation (*p* = 0.0027; *R*^2^ = 0.65) between cavity areas in OPL + HFL complex (mm^2^) and microperimetry 2 degree sensitivity (dB).

**Figure 7:**
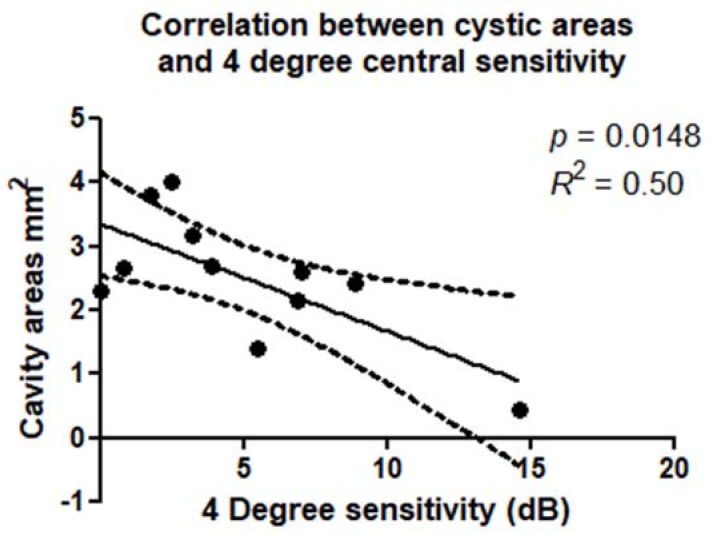
Scatterplot shows the statistically significant correlation (*p* = 0.0148; *R*^2^ = 0.50) between cavity areas on OPL + HFL complex (mm^2^) and microperimetry 4 degree sensitivity (dB).

There was a statistically significant correlation between retinal sensitivity and extension of the cystic cavities evaluated in the OPL + HFL complex by en face OCT. These correlations were confirmed within a 2° diameter (*p* = 0.0027; *R*^2^ = 0.65) (Figure 6) and 4° diameter (*p* = 0.0148; R^2^ = 0.50) around the MH (Figure 7). There was a statistically significant correlation between retinal sensitivity and area of no flow evaluated in the DCP on OCTA. These correlations were confirmed within a 2° diameter (*p* = 0.0031; *R*^2^ = 0.43) and 4° diameter (*p* = 0.0153; R^2^ = 0.64) around the MH.

In our study, we did not find any statistical correlation between 2° retinal sensitivity and the VD of the whole SCP (*p* = 0.52; *R*^2^ = 0.04) and the whole DCP (*p* = 0.23; *R*^2^ = 0.15). There was no statistical correlation between 2° retinal sensitivity and the VD of the inner SCP (*R*^2^ = 0.17; *p* = 0.2) and outer SCP (*p* = 0.98; *R*^2^ = 0.01), just as there was no statistical correlation between 2° retinal sensitivity and the VD of the inner (*p* = 0.08; *R*^2^ = 0.3) and outer DCP (*p* = 0.7; *R*^2^ = 0.02).

We did not find any significant correlation between 4° retinal sensitivity and the VD of the whole SCP (*p* = 0.21; *R*^2^ = 0.17) and the whole DCP (*p* = 0.13; *R*^2^ =0.24). There was no statistical correlation between 4° retinal sensitivity and the VD of the inner SCP (*R*^2^ = 0.04; *p* = 0.5) and outer SCP (*p* = 0.14; *R*^2^ = 0.24); similarly, there was no statistical correlation between 4° retinal sensitivity and the VD of the inner (*p* = 0.06; *R*^2^ = 0.3) and outer DCP (*p* = 0.62; *R*^2^ = 0.03).

**Figure 8:**
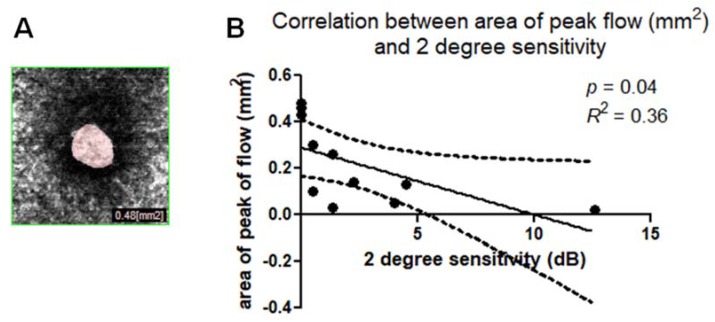
(**A**) Area of peak of flow in the choroidal slab (also named “choriocapillary transparency”) due to visibility of choroidal vessels caused by lack of neuroepithelium. (**B**) Scatterplot shows the statistically significant correlation (*p* = 0.04, *R*^2^ = 0.36) between the area of peak of flow in the choroidal slab (mm^2^) and microperimetry 2 degree sensitivity (DB).

The mean area of window defect (the area of peak flow on the choroidal slab due to a lack of neuroretinal tissue) was 0.22 ± 0.18 mm^2^. Linear regression analysis between the area of peak flow measured on the choroidal slab (also named “choriocapillary transparency”) (Figure 8A) and the 2° and 4° retinal sensitivity were plotted. A Pearson’s test indicated statistically significant correlation for 2° sensitivity, (*p* = 0.04, *R*^2^ = 0.36) (Figure 8B) and 4° sensitivity (*p* = 0.04, *R*^2^ = 0.36).

There was a statistically significant correlation between the postoperative BCVA and the preoperative extension of the cystic cavities (*p* = 0.03; *R*^2^ = 0.62), and between the postoperative BCVA and macular hole diameter (*p* < 0.001; *R*^2^ = 0.85). The larger MHs with a wider extension of cystic cavities were associated with lower postoperative BCVA. The postoperative BCVA was also correlated with the preoperative area of window defect (*p* = 0.003; *R*^2^ = 0.79) and to preoperative 2° retinal sensitivity (*p* = 0.02; *R*^2^ = −0.6).

## 4. Discussion

To our knowledge, this is the first report of a combined evaluation of structural findings (OCT, en face OCT) and functional parameters obtained by the integration of OCTA and microperimetry in patients with FTMH.

To date, most preoperative prognostic features identified for macular hole surgery have been proposed based on structural cross-sectional B-scan OCT findings, such as the minimum linear diameter, basal hole diameter, and the properties of the outer retinal layers [6].

Several previous studies [2] on FTMH have reported a significant decrease in BCVA due to tissue loss or displacement caused by vitreomacular traction, as measured as the hole diameter.

Although both the minimum diameter and basal hole diameter appear to be widely accepted as helpful for predicting visual outcomes following MH surgery, the results for these predictors vary across studies.

However, vision is also impaired by other factors, as documented in the current study. These factors provide interesting clues on the function of the affected retina during the natural history of FTMH. For example, visual impairment seems to be influenced by the anatomical condition of the area of the perilesional ring affected by large cystoid cavities. An interventional, retrospective case series evaluated photoreceptor inner/outer segment (IS/OS) defects, best-corrected visual acuity (BCVA), macular sensitivity, and fixation stability to correlate morphologic changes with visual functional outcomes at different stages after macular hole surgery using spectral-domain optical coherence tomography combined with microperimetry. Continuous anatomical and functional improvement was observed after successful macular hole surgery. The authors observed that the preoperative extent of the IS/OS junction defect is of good predictive value for postoperative macular sensitivity [21].

Another study [22] evaluated the correlations between anatomical and functional changes studied using microperimetry and spectral-domain OCT after the successful repair of idiopathic macular holes. In agreement with this, in our study, macular sensitivity (MS) and foveal sensitivity (FS) were lower, and the number of lesions of the outer retinal layers was higher in patients with a poorer postoperative VA. Preoperative MH size was lower and MS and FS were better in patients with a preserved junction line between the inner and outer segments of photoreceptors (IS/OS). Postoperative VA was significantly correlated with MS and FS. Postoperative outer retinal layer integrity was associated with better final retinal sensitivity.

Many studies have already used microperimetry to document the central absolute scotoma corresponding to the macular hole and a surrounding area of relative scotoma [23]. It is not novel that larger macular holes have larger scotomas, since, by definition, a macular hole is a defect in the neurosensory retina.

OCTA is a novel imaging technique that utilizes motion contrast to visualize retinal microvasculature in a rapid, noninvasive way. To date, several studies have reported vascular changes and quantitative characteristics of OCTA images in eyes with MH, considering this new technique to be a useful tool for the assessment of MHs before and after surgical treatment [24].

The segmentation is one of the main issues in OCTA interpretation, and in the case of FTMH, the automatic segmentation using the software may not be correct, due to the presence of cysts. A recent study described image artifacts that may occur in SS-OCTA imaging of full-thickness macular holes [25]. They described the “jellyfish-like” appearance which is visible in some patients with cystoid spaces at the margins of the macular hole. The striae are of similar reflectivity to retina tissue, and no flow is visible in the representation of “digestive canals.” Although they acknowledge the artifacts occurring during the visualization of FTMHs with SS-OCTA, the authors considered the role of OCTA in evaluating macular microcirculation in eyes with FTMHs before and after surgery and in the other eye of each patient. Thus, OCTA results must be evaluated very carefully.

The current study is focused on the use of microperimetry integrated with OCT angiography to evaluate the correlation between retinal structure and function in patients with idiopathic FTMH before surgery.

The microperimetry superimposed onto the en face layers and OCTA slabs shows the presence of an absolute central scotoma that corresponds precisely to the FTMH, which is in agreement with previous studies. Moreover, the combination of these techniques reveals that the absolute central scotoma corresponds to the choroid transparency visible on OCTA due to a complete lack of neuroretinal tissue. The automated overlay of retinal sensitivity maps with OCTA of the DCP and en face OCT showed that the perilesional ring of relative scotoma corresponded to the cysts, which were clearly defined on en face OCT as hyporeflective areas and in the OCTA scans of the DCP as areas of no flow.

We evaluated the cystic cavity extension in the OPL + HFL complex using en face OCT and only in DCP using OCTA, because the area of cystoid cavities can be underestimated if only the INL is considered, since the same measurement using OPL + HFL results in larger areas. In fact, in a recent study, we showed that the INL contained small, circular hyporeflective spaces surrounding the FTMH, and the OPL + HFL complex contained larger elongated radial hyporeflective cavities arranged in a stellate pattern. OCTA of the foveal region in FTMH was well-correlated with en face OCT scans, and the comparison of en face and OCTA images for perifoveal cystoid cavities associated with FTMH found statistically significant correlations between the two imaging modalities. In the same study, we observed that the mean cavity areas in deep vascular network were statistically significantly correlated between OCT-A and en face images. This observation implied that the retina surrounding the cystoid spaces preserved circulation, as shown by OCT-A. The overlap of images observed in en face and OCT-A indicated that OCT-A was able to detect the flow between one cystoid space and another. The residual flow suggested nonischemic tissue and “vascular sliding” at the border of the cavities. We hypothesize that this phenomenon could generate changes to the vascular structure causing retinal tissue damage.

In the present study, the microperimetry combined with OCTA was able to show areas of relative scotomas corresponding to the cystoid spaces; this could integrate the hypothesis of vascular slinding at the border of the cavities, with vascular alterations and consequent retinal tissue damage.

Moreover, the present study suggests that visual impairment, as measured as BCVA, correlates with the macular hole diameter (μm) and the cumulative cystic area, while the extension of the cysts correlates with the reduction in retinal sensitivity evaluated by microperimetry. This observation highlights the role of cystoid cavities in altering visual acuity. Moreover, the preoperative cumulative cystic area correlates with postoperative BCVA, suggesting that the larger MHs with a wider extension of cystic cavities were associated with lower pre- and postoperative BCVA.

Quantitative analysis of the overlap of microperimetry and en face images showed a statistically significant correlation between retinal sensitivity within the initial 2° and the cumulative cystic areas. The statistically significant correlation also held for the initial 4° in the current study.

The adjunctive role of perifoveal pseudocyst area as a prognostic factor in macular hole surgery has already been evaluated by a SD-OCT prospective study which highlighted that the anatomical closure was correlated with MH basal diameter and MH pseudocyst area. The authors concluded that perifoveal pseudocysts seem to be associated with anatomic failure, and may be useful as a prognostic factor in MH surgery.

Our current study has some limitations: above all, the small number of eyes and the lack of postoperative microperimetry evaluation. In fact, to confirm and integrate this data, it would be useful to evaluate FTMHs with the integration of OCTA, en face OCT and MP after surgery during follow-up, and extend the analysis to FTMHs associated with other retinal disease. Moreover, in a further study, it would be interesting to analyze a potential correlation between the OCTA area of no flow and the OCT size of cystic cavity.

In conclusion, our study shows that retinal sensitivity is related to the diameter of the FTMH, the width of the perifoveal cystic cavities, and the area of choroidal trasparency.

Although the most important surgical prognostic factors of MHs are based on cross-sectional OCT images, it could be useful to include other parameters from other imaging techniques (OCTA) to evaluate patients affected by MH before surgery and correlate them to functional findings obtained using functional techniques such as microperimetry.

## 5. Conclusions

OCT and OCTA combined with microperimetry may be useful to evaluate the morphologic and functional aspects of a macular hole. The integration of structural and functional techniques provides novel data that correlate function via microperimetry and structure through OCT imaging, which may be important to evaluate pre- and post- operative function. Moreover, it may be useful to evaluate the morphofunctional restoration by both microperimetry and OCTA after FTMH surgery.

This integrated evaluation of FTMHs and the noninvasive assessment of vascular involvement would be a useful tool to identify changes in microcirculation before and after vitreoretinal surgery, correlating them to functional changes in order to study new potential prognostic factors.

The use of integrated microperimetry may serve to analyze the functional consequences of the morphological alterations and assess the outcomes and treatment benefits in a more objective way.

Additional multicenter studies enrolling larger sample sizes are warranted to verify the preliminary findings reported here.
